# Virus‐like particles: Next‐generation nanoparticles for targeted therapeutic delivery

**DOI:** 10.1002/btm2.10049

**Published:** 2017-01-19

**Authors:** Marcus J. Rohovie, Maya Nagasawa, James R. Swartz

**Affiliations:** ^1^ Dept. of Chemical Engineering Stanford University Stanford CA 94305; ^2^ Dept. of Bioengineering Stanford University Stanford CA 94305

**Keywords:** nanoparticle, protein engineering, virus‐like particle, targeted delivery, therapeutics

## Abstract

Most drug therapies distribute the agents throughout the entire body, even though the drugs are typically only needed at specific tissues. This often limits dosage and causes discomfort and harmful side‐effects. Significant research has examined nanoparticles (NPs) for use as targeted delivery vehicles for therapeutic cargo, however, major clinical success has been limited. Current work focuses mainly on liposomal and polymer‐based NPs, but emerging research is exploring the engineering of viral capsids as noninfectious protein‐based NPs—termed virus‐like particles (VLPs). This review covers the research that has been performed thus far and outlines the potential for these VLPs to become highly effective delivery vehicles that overcome the many challenges encountered for targeted delivery of therapeutic cargo.

**Abbreviations**
CCMV: cowpea chlorotic mottle virus
CPMV: cowpea mosaic virus
EDC: 1‐ethyl‐3‐(3‐dimethylaminopropyl)carbodiimide hydrochloride
EGF: epidermal growth factor
EPR: enhanced permeability and retention
HBVc: hepatitis B virus core
MS2: enterobacteria phage MS2
NHS: n‐hydroxysuccinimide
NP: nanoparticle
P22: *Salmonella typhimurium* P22
PEG: polyethylene glycol
Qβ: enterobacteria phage Qβ
VLP: virus‐like particle

## 1 Introduction

Currently, numerous diseases lack adequate treatment, most notably cancer and various genetic disorders. In 2016, the National Cancer Institute estimates that 1,685,210 new cases of cancer will be diagnosed in the United States and 595,690 people will die from the disease—a 35% mortality rate.^1^ Typical cancer treatment includes chemotherapy, radiation, and surgery. However, surgery is highly invasive and often fails—especially after metastasis. Chemotherapy and radiation can be effective temporarily, but result in harsh side‐effects that drastically reduce quality of life. In particular, systemic administration of chemotherapeutic agents is usually limited by those side‐effects and the effective dose at the tumor site is only a small fraction of the administered drugs.^2^

In addition to cancer, the development of gene therapies for treatment of genetic disorders, such as mitochondrial disorders and Parkinson's disease has been a major focus of research. By 2012, over 1800 gene therapy clinical trials had been conducted or approved.^3^ However, success in clinical trials has been limited because of numerous technical barriers, including difficulty in reaching the targeted tissues. Although the U.S. FDA approved the first oncolytic viral therapy, Imlygic, recently, no virus‐derived therapies for gene delivery have been FDA‐approved according to the latest information from the FDA's website.

Targeted delivery has long been one of the most promising, but also most challenging, opportunities for improving the treatment of these diseases. Targeted delivery offers three key advantages that systemic delivery lacks: (a) the therapeutic will act primarily at the desired site‐of‐action, limiting off‐target effects such as the harmful side‐effects involved with chemotherapy; (b) the delivery vehicle can provide much higher local concentrations of the therapeutic within the diseased tissues, allowing a more effective treatment; and (c) the delivery vehicle can carry the therapeutic to sites it would not normally be able to reach, improving the efficiency of gene therapies. The first attempts at developing delivery vehicles were antibody‐drug conjugates. These carriers have been extensively developed with two FDA‐approved examples [Trastuzumab emtansine (Kadcyla) and Brentuximab vedotin (Adcetris)], and many more are in clinical trials.^4^ However, they suffer from several limitations including structural heterogeneity, instability, and limited solubility.^4^, ^5^ In addition, antibody‐drug conjugates typically deliver only a few drug molecules per antibody.^4^ In contrast, nanoparticle (NP)‐based delivery agents, including liposomal, polymer‐based, metal‐based, and protein‐based NPs, have the potential to provide safer and more effective delivery by encapsulating therapeutic cargo inside the particle with a much higher cargo/carrier ratio. This review will focus on the development of virus‐like particles (VLPs), protein‐based NPs derived from viral capsids, as targeted therapeutic delivery agents. Several previously published reviews have covered VLPs. A review from the Bundy lab excellently describes many ways to covalently attach ligands to the surface of VLPs, but lacks further information pertinent to their use as drug delivery vehicles.^6^ A 2014 review from the Tullman‐Ercek lab covers cargo loading, specific targeting, and application for using VLPs as delivery vehicles, but lacks specific surface modification information and loading small molecule drugs.^7^ Another 2014 review from the van Hest lab has an excellent discussion of surface and interior covalent attachment and genetic fusion strategies, but contains no discussion of nonspecific cargo loading or attachment techniques.^8^ Lastly, a recent 2016 review covers a large variety of VLPs and other protein‐based NPs, but lacks depth for each individual vehicle.^9^ This review, while focusing on six of the most used VLPs, attempts to combine and expand on the information within these other reviews while addressing new factors, including particle stability, expression platforms, and purification methods, that are important for the development of these vehicles as therapeutic NPs.

## 2 Using vlps overcomes the limitations of current np‐based therapeutics

Despite many attempts, only a few liposomal and protein‐based NPs have been approved for cancer drug delivery, including Doxil and Abraxane—and these are all passive‐targeting delivery agents relying on the enhanced permeability and retention (EPR) effect for tumor localization.^5^, ^10^, ^11^ Liposomal NPs are limited by particle instability, rapid clearance, and spontaneous membrane fusion with off‐target cells.^12^, ^13^ The polymer‐based NP technologies suffer from structural heterogeneity, particle instability, slow and nonuniform drug release, and potential immunogenicity.^14^, ^15^ The more stable metal‐based NPs suffer from a lack of specificity and high toxicity.^16^ In addition, most of these NPs suffer from clearance mediated by phagocytes and dendritic cells, including Kupffer cells in the liver. Coating NPs with polyethylene glycol (PEG) can help avoid phagocytes and extend the blood circulation time by creating “stealth” brushes.^17^, ^18^ However, PEGylation can also reduce NP uptake by the targeted cells and is potentially immunogenic.^17^, ^18^ Finally, surface functionalization of these NPs is difficult to control and nonuniform.^19^

An alternate type of drug delivery NP that is showing promise is the VLP.^20^ VLPs are self‐assembled, homogeneous NPs derived from the coat proteins of viral capsids. They typically lack their natural genome and are therefore noninfectious. VLPs are an emerging class of targeted delivery vehicles with the potential to overcome the limitations of other NPs.^7^, ^20^ In recent years, several groups have shown that VLPs can pack and deliver therapeutic cargo such as chemotherapeutic drugs, siRNA, RNA aptamers, proteins, and peptides.^21^, ^22^, ^23^, ^24^, ^25^, ^26^, ^27^ However, there are still challenges when using VLPs. Similar to other NPs, avoiding phagocyte‐mediated clearance is a major challenge, even with PEGylated VLPs.^22^, ^28^ In addition, VLP stability can also be an issue.^29^ Lastly, recent research has shown that ellipsoid NPs are able to extravasate from the blood vessel more effectively than spherical NPs.^30^ This ellipsoid shape is possible for conventional polymeric NPs, but is not feasible for icosahedral VLPs. However, the capability of VLP surface modification allows a variety of functional ligands to be added with the potential to address these limitations. By displaying multiple ligands with high affinity for the tight junctions between endothelial cells, VLPs may be able to efficiently extravasate from the vasculature of the blood vessels.

## 3 Challenges to targeted delivery using NPs

As mentioned previously, targeted drug delivery by NPs must overcome multiple challenges (Table 1).^31^ The ability to overcome these challenges must either be intrinsic or be imparted as the VLPs are prepared by: (a) loading the cargo inside the NP, and (b) functionalizing the NP to deliver its cargo primarily to the intended cells. While in the bloodstream and the interstitial space, the NP must remain stable, retain its cargo, and avoid nonspecific engulfment by phagocytes. Additionally, after extravasation into the extravascular tissue, the NP must specifically target the intended cells while avoiding other healthy cells to limit organ accumulation and toxicity. After adsorption to the targeted cells and internalization through receptor‐mediated endocytic pathways, NPs carrying macromolecular cargo must escape the endosome, disassemble, and release their therapeutic cargo to the cytosol (in a functional form). Even though endosomal escape may not be required when delivering small, stable molecules (since subsequent lysosomal degradation of the NP should eventually release the cargo), these other requirements still present a daunting challenge for the development of targeted delivery vehicles. However, combining surface functionalization of the VLPs with the ability to load therapeutic cargo can provide the design flexibility and complexity needed to open the door to multiple new therapies for unmet medical needs. The several attempts to overcome these challenges are outlined in the following sections.

**Table 1:** Challenges to targeted drug delivery and possible solutions

Challenge	Possible solutions	Tested VLPs
*Stabilize nanoparticle*	Stabilize with disulfide bonds	HBVc, MS2, Qβ
*Avoid phagocytes*	Display PEG or the CD47 ectodomain	MS2, Qβ, P22, CCMV, CPMV
*Extravasate from blood vessel*	—	—
*Target specific cells*	Display targeting ligands	HBVc, MS2, Qβ, CPMV
*Escape endosome*	Display cell‐penetrating peptides	MS2, P22, CPMV
*Release cargo*	Reduce stabilizing disulfide bonds in cytosol	HBVc, Qβ

## 4 Commonly used vlps and their production methods

**Figure 1:**
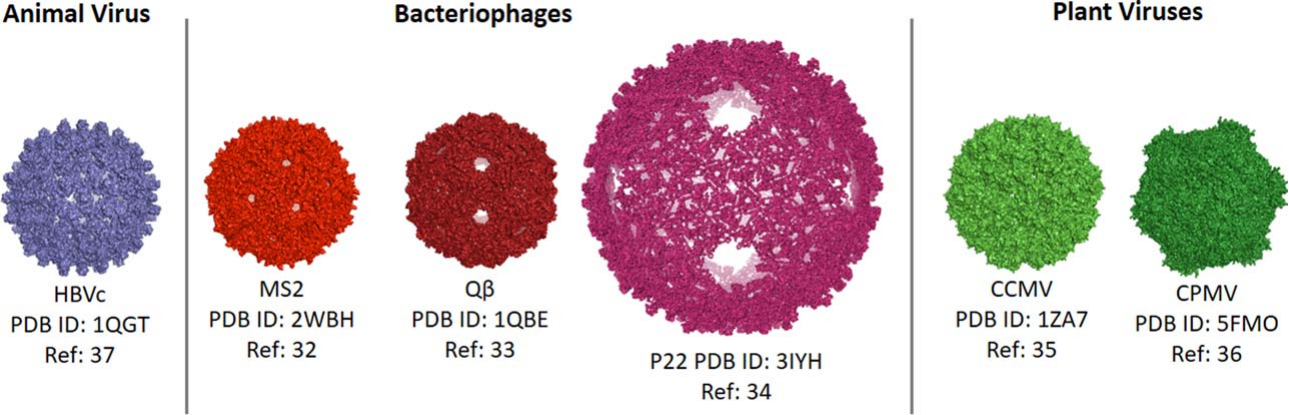
Structures of the six VLPs discussed in this review

This review will focus on six of the most actively developed VLPs from: one animal virus, three bacteriophages, and two plant viruses (Figure 1).^32^, ^33^, ^34^, ^35^, ^36^, ^37^ Table 2 summarizes the properties of these VLPs.

**Table 2:** Relevant information on the six VLPs discussed in this review

VLP	Virus type	VLP outer diameter (nm)	VLP inner diameter (nm)	VLP geometry	VLP subunits	References
*HBVc*	Animal virus	35	26	*T* = 4 icosahedral	240 coat proteins (120 dimers)	37, 40, 48
*MS2*	Bacteriophage	27	15	*T* = 3 icosahedral	180 coat proteins (90 dimers)	38, 42, 49
*Q*β	Bacteriophage	28	21	*T* = 3 icosahedral	180 coat proteins (90 dimers)	39, 43
*P22*	Bacteriophage	58‐64	48–50	*T* = 7 icosahedral	420 coat proteins + 100–300 removable scaffold proteins	41, 50
*CCMV*	Plant virus	28	18	*T* = 3 icosahedral	180 coat proteins (90 dimers)	51, 52
*CPMV*	Plant virus	28–31	22	*T* = 3 icosahedral	60 large + 60 small coat proteins	53, 54

### 4.1 Animal virus‐based VLPs

The hepatitis B virus is comprised of an internal protein capsid and a lipid envelope containing other proteins. Two different VLPs can be produced from the virus, using either the core antigen that forms the internal capsid or the surface antigen that spontaneously combines with lipids to form NPs. We will focus on the VLP derived from the hepaitis B core (HBVc) antigen, which is formed from 240 copies of a single protein.^37^ These proteins first form dimers, which then assemble with pentameric or pseudo‐hexameric junctions in a *T* = 4 icosahedral geometry.^37^, ^38^, ^40^, ^48^ The VLP has been produced using multiple technologies including *Escherichia coli* cytosolic accumulation and cell‐free protein synthesis.^37^, ^38^ The assembled VLPs are typically purified using size‐exclusion chromatography or differential centrifugation.^37^, ^38^, ^49^ Individual coat proteins have been subsequently obtained by disassembling the VLPs with urea, which allows simultaneous cargo loading and VLP re‐assembly.^37^, ^38^, ^49^ Unpublished data from the Swartz group indicates that coat proteins with hexahistidine extensions can also be directly purified using immobilized metal affinity chromatography. Purifying the individual coat proteins allows control over cargo loading during VLP assembly, which in the case of HBVc is achieved by increasing the salt concentration to trigger spontaneous self‐assembly mediated primarily by hydrophobic interactions.^37^, ^38^

### 4.2 Bacteriophage‐based VLPs

The three bacteriophages, MS2, Qβ, and *Salmonella typhimurium* P22, all infect enterobacteria, most notably *E. coli*. Although all three are composed of only a nucleic acid‐filled viral capsid, P22 differs greatly from MS2 and Qβ. MS2 and Qβ are composed of 90 homodimers and require a specific stem‐loop hairpin secondary structure in their RNA genome to trigger VLP self‐assembly by binding to the coat proteins.^38^, ^39^, ^42^, ^43^ P22, on the other hand, is composed of up to 415 coat proteins, 100–300 scaffold proteins, and 12 portal proteins. However, the P22 VLP has been engineered to consist of 420 coat proteins and only the 100–300 scaffold proteins, which can subsequently be removed with guanidine hydrochloride, leaving only the coat proteins.^41^, ^50^ Like the HBVc VLP, these VLPs assemble with icosahedral geometry.^38^, ^41^, ^43^, ^51^, ^52^, ^53^ All three can be produced in *E. coli*, but Qβ can also be produced in yeast and both Qβ and MS2 can be produced using cell‐free protein synthesis.^29^, ^38^, ^41^, ^43^, ^50^, ^54^, ^55^, ^56^ MS2 VLPs have been purified using size‐exclusion chromatography, differential centrifugation, or immobilized metal affinity chromatography (for VLPs containing hexahistidine tags).^38^, ^44^, ^54^ Acids or urea can be used to disassemble the purified MS2 VLPs to obtain the dimers, which can then be re‐assembled after removal of the disassembly agent and the addition of the stem‐loop RNA.^23^, ^42^, ^45^ Qβ VLPs have been purified using size‐exclusion chromatography and the dimers can be obtained by disassembling the VLPs using acid, which can then be reassembled similar to MS2.^39^, ^43^, ^57^ P22 VLPs have been purified using size‐exclusion chromatography or differential centrifugation and can also be disassembled using acid to obtain the coat proteins.^41^, ^50^, ^55^, ^56^ Addition of scaffold proteins is required to reassemble the P22 VLP, but these can subsequently be removed.^41^ These bacteriophage‐derived VLPs differ from HBVc VLPs mainly in the assembly stimulus, using additional biomolecules (RNA or proteins) to initiate self‐assembly instead of increasing the salt concentration.

### 4.3 Plant virus‐based VLPs

The final two commonly‐used VLPs to be discussed are derived from plant viruses that infect the cowpea leaf: cowpea chlorotic mottle virus (CCMV) and cowpea mosaic virus (CPMV). Neither virus has a lipid envelope. Both VLPs assemble with icosahedral geometry.^46^, ^47^, ^58^, ^59^, ^60^ The CCMV VLPs are formed from 90 homodimers and can be produced in *E. coli* or yeast.^46^, ^61^ They have been purified using size‐exclusion chromatography or immobilized metal affinity chromatography, using coat proteins with hexahistidine extensions.^46^, ^62^ Dimers can be obtained by dialyzing the assembled VLPs against 0.5 M CaCl_2_ or by purifying hexahistidine tagged dimers directly.^47^, ^62^ Combining the dimers with RNA in a 1:6 mass ratio and lowering the pH to 4–5 induces self‐assembly.^47^, ^62^, ^63^, ^64^ CPMV, on the other hand, is formed from 60 copies of the VP60 protein which must first be proteolyzed into the L and S coat proteins (60 copies of each).^59^ Unfortunately, the VLP cannot be produced using *E. coli* or yeast; insect cells or plants must be used.^58^, ^59^ The VLPs have been purified using differential centrifugation, but the coat proteins cannot yet be obtained in usable quantities.^58^, ^59^ The inability to produce the VLP in *E. coli* or obtain purified coat proteins adds another challenge for targeted drug delivery; however, CPMV has been actively evaluated for therapeutic use due to the ability to easily display ligands on its surface and load cargo through association with its genome.

## 5 Design considerations in developing VLPs for targeted delivery

Because of their precise and repeated structures and relatively large cargo capacities, VLPs have many advantages over other types of NPs. Since they are expressed biologically and formed from multiple copies of the same protein, the VLPs are highly uniform and are easily expressed in bacteria (with some exceptions, such as CPMV). Also, they have evolved in nature to encapsidate their viral genomes, which could be advantageous for loading therapeutic cargo. Particularly in the case of MS2 and Qβ, specific stem‐loop RNA secondary structures that are required for assembly can carry other molecules into the VLP during assembly.^21^, ^42^, ^65^, ^66^ For many VLPs, peptide or protein sequences can be added directly to the primary amino acid sequence of the coat proteins either as insertions or extensions to allow presentation on either the interior or exterior surface.^67^, ^68^, ^69^, ^70^, ^71^ Alternatively, reactive amino acids can be used to couple cargo to the interior or ligands to the exterior of the capsids in repeated and consistent orientations.^66^, ^72^, ^73^, ^74^ VLPs are much less toxic for parenteral administration than metal NPs, more stable than liposomes, and more uniform than polymer NPs.^75^, ^76^, ^77^ Although a significant amount of work is still required to develop the VLPs as delivery vehicles, the current progress shows a great deal of promise.

### 5.1 Surface functionalization

**Figure 2:**
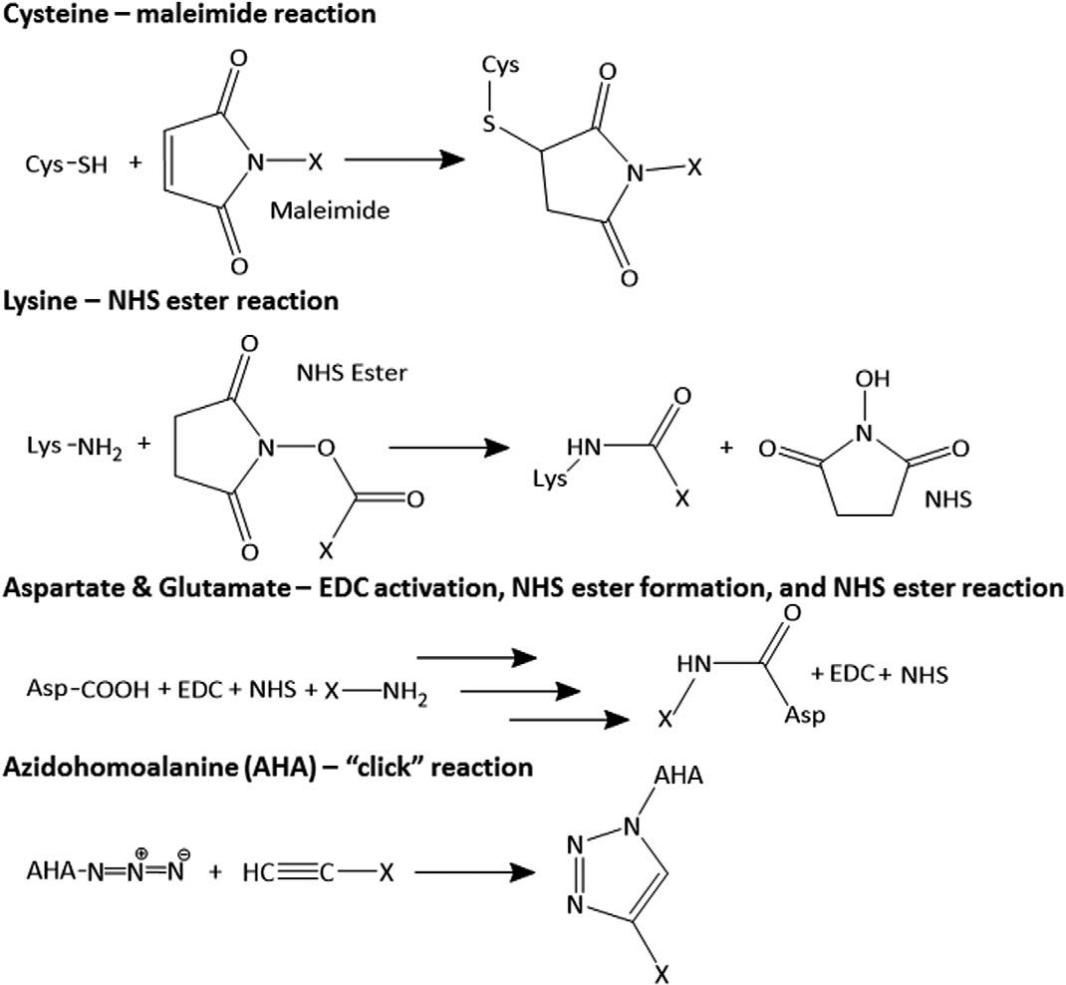
Common conjugation chemistries. Reactions used to functionalize the exterior and interior of VLPs at reactive amino acids (X is a ligand or cargo)

As discussed, a delivery vehicle must provide several different functionalities. For several of these attributes, the VLP surface must be extensively modified with various biomolecules. These ligands can provide specific cellular targeting, reduce immune responses, and potentially facilitate extravasation. Most approaches require covalent attachment, however, P22 can display ligands through noncovalent interactions.^78^ Covalent methods take advantage of either native or nonnatural reactive amino acids (Figure 2), though genetic fusions to the primary amino acid sequence can also be used to display inserted peptides or proteins.^67^, ^68^, ^69^, ^70^, ^71^ Table 3 provides a summary of these surface modifications, including specific references. Although many of the published surface modifications are aimed at vaccines or other uses not related to drug delivery, the same methods can easily be applied. Furthermore, some published studies have described the attachment of ligands for targeting specific cells or avoiding the immune system.^78^, ^84^, ^85^, ^86^ These will be discussed further in later sections.

**Table 3:** Surface ligands displayed on the VLPs

VLP	Surface functionalization	Method	References
*HBVc*	Antibody fragment	Genetic fusion to coat protein	67
	Green fluorescent protein	Genetic fusion to coat protein	67
	Flagellin	“Click” chemistry	101
*MS2*	Antibody fragment	“Click” chemistry	79
	Transferrin	Conjugated to surface lysines	66
	DNA aptamers	paF‐based oxidative ring contraction	84,89,100
	Granulocyte macrophage colony‐stimulating factor	“Click” chemistry	79
	Nucleic acids	“Click” chemistry	79
	PEG	“Click” chemistry	79
		paF‐based oxidative ring contraction	89,121
	Foreign epitopes (as a selection screen)	Genetic fusion to coat protein	68
	HIV‐Tat cell‐penetrating peptide	Conjugated to surface cysteines	90,95,102
*Q*β	Glycans	“Click” chemistry	85
		Conjugated to surface lysines	52,142
	Human epidermal growth factor	Genetic fusion to coat protein	69
	Antibody fragment	“Click” chemistry	79
	Transferrin	“Click” chemistry	141
	Ganglioside GM2 tumor‐associated carbohydrate antigen	“Click” chemistry	91
	Metalloporphyrin derivative	“Click” chemistry	142
	Granulocyte macrophage colony‐stimulating factor	“Click” chemistry	79
	Nucleic acids	“Click” chemistry	79
	PEG	“Click” chemistry	79
*P22*	CD47 “self‐peptide”	Genetic fusion to “decoration protein”	78
	CD40L	Genetic fusion to “decoration protein”	78
	HIV‐Tat cell‐penetrating peptide	Conjugated to surface cysteines	24
	Peptide tags (for further modification)	Genetic fusion to coat protein	70
	MIANS (fluorescent probe)	Conjugated to surface cysteines	74
*CCMV*	Foreign epitope (S9 peptide)	Conjugated to surface cysteines	92
	Alkynes	“Click” chemistry	105
	PEG	Conjugated to surface lysines	98
	Biotin	Conjugated to surface lysines	99
	Fluorescent probes	Conjugated to cysteines, lysines, aspartates, or glutamates	72
	Peptides	Conjugated to cysteines, lysines, aspartates, or glutamates	72
*CPMV*	RGD peptide (integrin‐binding)	“Click” chemistry	96
		Conjugated to surface lysines	96
	Pan‐bombesin analogue (with fluorescent probes and PEG)	“Click” chemistry	58
	Glycans	“Click” chemistry	85
		Conjugated to surface lysines	94
	Folic acid‐PEG	“Click” chemistry	86
	Foreign epitope (peptide antigens)	Genetic fusion to coat protein	71
		Conjugated to surface cysteines	92
	Fluorescent probes	Conjugated to surface cysteines or lysines	73,82,135
	PEG	Conjugated to surface lysines	82,135
	R5 cell‐penetrating peptides	Conjugated to surface lysines	80
	VEGFR‐1 ligand	Conjugated to surface lysines	81
	Gd‐DOTA	“Click” chemistry	103,104
	Heterologous proteins	Conjugated to surface cysteines or lysines	93

#### 5.1.1 Cysteine‐based modifications

Arguably the most commonly used reactive amino acid residue, cysteine, can be presented either naturally or by mutation on the VLP surface. Because of its free sulfhydryl group, cysteine will readily and spontaneously form disulfide bonds with other sulfhydryl‐containing ligands under oxidative conditions. However, the disulfide bond is also easily reduced and may not be ideal for surface attachments. Alternatively, a series of compounds based on maleimide readily and irreversibly form thioether linkages with cysteine residues at a pH between 6.5 and 7.5. This attachment strategy has been used to display cell‐penetrating peptides, fluorescent probes, and heterologous peptides and proteins on the surfaces of MS2, P22, CCMV, and CPMV VLPs.^24^, ^70^, ^72^, ^73^, ^74^, ^90^, ^92^, ^93^, ^95^, ^102^

#### 5.1.2 Lysine‐based modifications

Another common amino acid residue that is easily modified is lysine because of its primary amine. Using reactions termed *n*‐hydroxysuccinimide (NHS) ester reactions (because NHS is released as part of the reaction), amide bonds are formed at surface‐exposed lysine residues. The reaction occurs spontaneously between pH 7.2 and 9. This attachment chemistry has been used to display transferrin on MS2, which may allow the VLP to transcytose the blood‐brain barrier, a development that could open up a new library of therapies for neurological disorders.^66^ Additionally, PEG, peptides, other proteins, and fluorescent probes have been displayed on Qβ, CCMV, and CPMV VLPs using the NHS reaction.^72^, ^73^, ^80^, ^81^, ^82^, ^88^, ^94^, ^95^, ^98^, ^99^

#### 5.1.3 Aspartate‐ or glutamate‐based modifications

Although not as commonly used, the last class of reactive natural amino acid residues includes the carboxylic acids aspartate and glutamate. Unlike strategies involving cysteine and lysine, coupling to these residues requires multiple steps. First, the carboxylic acid must be activated using 1‐ethyl‐3‐(3‐dimethylaminopropyl)carbodiimide hydrochloride (EDC). Once activated, it will react with NHS to form an NHS ester. Now that the carboxylic acid side‐chain has essentially become an NHS ester, previously described with regard to lysine modifications, we can use a ligand with an exposed primary amine to form a stable amide bond. This strategy has been used primarily with CCMV to display peptides or fluorescent probes.^72^

#### 5.1.4 Nonnatural amino acid‐based modifications

Beyond the 20 natural amino acids, many nonnatural amino acids have been used for site‐specific protein conjugation reactions. The two nonnatural amino acids most frequently incorporated into VLP coat proteins are azidohomoalanine (AHA) and *p*‐amino‐phenylalanine (pAF). These amino acids are incorporated into proteins in two ways: global methionine replacement and amber stop codon suppression. Because AHA is very similar to methionine, AHA will be incorporated at each AUG codon if the methionine supply is rate‐limiting; this is termed global methionine replacement.^79^ Bacteria auxotrophic for methionine or cell‐free protein synthesis can be used to limit methionine availability.^38^ The protein yield using global methionine replacement can be rather high from optimized procedures, but this approach will not work for pAF. Amber stop codon suppression, although more difficult and providing lower yields, will incorporate pAF. Amber stop codon suppression uses nonnative synthetases and tRNAs that do not react with the natural amino acids to incorporate the nonnatural amino acid at the amber stop codon UAG.^84^, ^89^, ^100^ However, the release factor protein for the amber stop signal is still present, so premature termination of the protein may also occur. Cell‐free protein synthesis offers a definite advantage here since optimized concentrations of the synthetic components can easily be added to the reaction, but premature termination still usually limits product accumulation.

Despite the drawbacks, these methods have been used to incorporate nonnatural amino acids with uniquely reactive side‐chains. AHA, displaying an azide, will participate in copper(I)‐catalyzed azide‐alkyne cycloaddition (“click” reaction) and form covalent triazole rings with alkyne‐containing ligands.^79^ This method has been used to display antibody fragments, folic acid, and RGD peptides on MS2 and CPMV, all of which have been shown to allow selective targeting of cancer cells.^80^, ^86^, ^96^ Additionally, heterologous proteins, peptides, nucleic acids, and PEG have been displayed on HBVc, MS2, Qβ, CCMV, and CPMV VLPs using this approach.^79^, ^85^, ^91^, ^101^, ^103^, ^104^, ^105^ pAF has been incorporated into MS2 and conjugated to ligands displaying phenylene diamines and aminophenols.^84^, ^89^, ^100^

#### 5.1.5 Genetic modifications

The final covalent attachment method we will discuss is genetic modification, in which the gene for the desired surface ligand is fused to the gene for the coat protein of the VLP. While the added peptide can inhibit protein folding as well as VLP assembly, this approach has been shown to work for most VLPs, but not CCMV.^67^, ^68^, ^69^, ^70^, ^71^ Most of the work has fused proteins to either termini of the coat protein, but the HBVc VLP has also been shown to accept heterologous protein domains within the sequence of the coat protein itself.^67^ As shown in Figure 1, the HBVc VLP possesses 120 “spikes” on its surface. Protein domains have been inserted such that they are displayed at each spike, allowing optimal surface presentation. These genetic fusion methods have been used to display various heterologous proteins including antibody fragments for specific cellular targeting.^67^

#### 5.1.6 Affinity‐based noncovalent modifications

P22 is unique compared to the other VLPs because of the existence of the decoration (or “dec”) protein. This protein has high affinity for the surface of P22.^78^ By fusing ligands to the “dec” protein, an affinity‐based noncovalent system was developed for surface display on the P22 VLP without requiring alteration of the coat proteins. This approach was used to display CD40L, derived from T cells, and the CD47 “self‐peptide,” developed by the Discher lab, which shows promise in avoiding phagocyte engulfment.^106^

### 5.2 Efficient cargo loading and retention

VLPs have been used to load a range of molecules, including small molecules (chemotherapeutics, fluorescent probes, polymers), nucleic acids, peptides, proteins, and even other NPs.^21^ The approaches include both covalent and noncovalent methods. Noncovalent methods are ideal as they do not require modification of the cargo, however, covalent methods typically have the advantage of more efficient encapsidation and retention of the cargo. As with surface modifications, most covalent methods for cargo loading take advantage of reactive amino acids and use the same chemistries described above (Figure 2), though some use genetic fusions to the primary amino acid sequence.^25^, ^83^, ^107^ Both methods will be discussed for the different types of cargo. See Table 4 for a list of cargo and loading methods, including specific references.

**Table 4:** Cargo loaded by the VLPs

VLP	Cargo	Method	References
*HBVc*	RNA (viral, heterologous)	Electrostatic adsorption	111,112
	DNA (CpG, single‐stranded, double‐stranded)	Electrostatic adsorption	65, 111, 113
	Green fluorescent protein	Passive encapsidation	116
	Nuclease	Genetic fusion to coat protein	107
	Iron oxide NP (IONP)	Hexahistidine:NTA coordination	49
*MS2*	Taxol	Conjugated to surface cysteines	114
	Alexa Fluor® 488	Conjugated to interior cysteines	84, 89
	Porphyrin	Conjugated to interior cysteines	100
	Doxorubicin	Conjugated to stem‐loop RNA	21
	Fluorescein	Conjugated to interior tyrosines	115
	DOTA chelators	Conjugated to interior cysteines	89, 121
	RNA (messenger, micro, small‐interfering)	Genetic fusion to stem‐loop RNA	21, 42, 66, 90, 102, 95
	Ricin toxin A‐chain	Conjugated to stem‐loop RNA	21
	HIV‐1 Tat peptide	Conjugated to stem‐loop RNA	118
	Alkaline phosphatase	Electrostatic attraction to coat protein	23, 119
	Green fluorescent protein	Electrostatic attraction to coat protein	23
	Quantum dot 585	Conjugated to stem‐loop RNA	21
*Q*β	Methacrylate (monomers, polymers)	“Click” chemistry	22
	CpG DNA	Electrostatic attraction to coat protein	65
	Fluorescent proteins	Adsorption to extension on stem‐loop RNA	52
	Luciferase	Adsorption to extension on stem‐loop RNA	39
*P22*	Nickel	Conjugated to interior cysteines	41
	Biotin	Conjugated to interior cysteines	124
	Fluorescein polymethacrylate	Conjugated to interior cysteines	125
	Gadopentetic acid polymethacrylate	Conjugated to interior cysteines	125
	CRISPR (Cas9 and guide RNA)	Genetic fusion to scaffold protein	25
	Green fluorescent protein or mCherry	Genetic fusion to scaffold protein	120, 123
	CellB protein	Genetic fusion to scaffold protein	120
	[NiFe] hydrogenase	Genetic fusion to scaffold protein	117
	Ziconotide peptide	Genetic fusion to scaffold protein	24
	Three enzyme cascade (genetically linked)	Genetic fusion to scaffold protein	122
	Alcohol dehydrogenase	Genetic fusion to scaffold protein	127
*CCMV*	Polystyrene sulfonate	Electrostatic adsorption	98, 126
	RNA	Electrostatic adsorption	64
	Green or teal fluorescent Protein	Genetic fusion	62, 83, 128
		Attraction between “leucine zipper” domains	62, 83, 128
	Pseudozyma antarctica lipase B	Attraction between “leucine zipper” domains	83
	Horseradish peroxidase	Passive encapsidation	46
	DOTAC10 micelles with Gd(III) or Zn(II)	Electrostatic adsorption	130
	Gd(DOTA)	“Click” chemistry	104
*CPMV*	Fluorescent probes	Conjugated to interior cysteines	108
	Doxorubicin	Conjugated to surface aspartates or glutamates	109
	DAPI	Electrostatic adsorption	110
	Acridine orange	Electrostatic adsorption	110
	Propidium iodide	Electrostatic adsorption	110
	Proflavin	Electrostatic adsorption	110
	Iron oxide NP	Passive encapsidation	129
	Gd(III)	Coordinated by genomic RNA	103, 104
	Tb(III)	Coordinated by genomic RNA	103, 104

#### 5.2.1 Small molecules

Unfortunately, there has been no published work describing the loading of small molecules within the HBVc VLP. The bacteriophages, on the other hand, have been used extensively to load small molecules. MS2 has been shown to encapsidate chemotherapeutics (taxol and doxorubicin), fluorescent probes (Alexa Fluor® 488 and fluorescein), and other small molecules (porphyrin and DOTA chelators) through conjugations to interior cysteines (disulfide‐ and maleimide‐based linkages), interior tyrosines (diazonium coupling), and the stem‐loop RNA hairpin required for VLP assembly.^21^, ^84^, ^89^, ^100^, ^114^, ^115^, ^121^ Qβ VLPs with AHA incorporated using global methionine replacement have been shown to covalently conjugate methacrylate to the coat proteins using the “click” reaction and encapsidate them during VLP assembly.^22^, ^79^ Lastly, the P22 VLP has been shown to covalently load nickel ions (through iodo‐phen linkages) and derivatives of biotin, fluorescein, and gadopentetic acid (through maleimide‐based initiators) by conjugating to interior cysteines.^45^, ^124^, ^125^

The plant virus‐based VLPs are unique compared to the others because they allow noncovalent loading of small molecules. CCMV has been shown to load polystyrene sulfonate through noncovalent electrostatic interactions between the cargo and the coat proteins.^98^, ^126^ CPMV has been used to covalently load chemotherapeutics (doxorubicin) through conjugation to aspartates and glutamates (EDC/NHS reactions followed by esterification) and maleimide derivatives of fluorescent probes by attachment to cysteine residues.^108^, ^109^ Additionally, fluorescent probes and an antibiotic (proflavin) have been noncovalently incorporated within CPMV with the small molecules electrostatically adsorbed by CPMV's RNA genome.^110^

Although these molecules may not all be therapeutically relevant, the results indicate that the methods can successfully load small molecules into many of the VLPs and the approaches can easily be extended to load other small molecule drugs. However, the fact that the only noncovalent loading of small molecules uses a polymerized cargo (that is bigger than most chemotherapeutics) or adsorbs the molecule within nucleic acids shows how difficult it is to load and retain these small cargoes. This is due to the presence of pores throughout the VLP structures, as seen in Figure 1. The development of nonporous VLPs would allow more efficient noncovalent loading and retention of drugs and will be beneficial for future drug delivery strategies using VLPs.

#### 5.2.2 Nucleic acids

MS2 VLPs are particularly suited to loading RNA. They require a short stem‐loop RNA hairpin, which is typically part of their genomic RNA, to assemble into capsids.^42^ This sequence has been easily extended to incorporate mRNAs, micro RNAs, and small interfering RNAs.^21^, ^42^, ^66^, ^90^, ^95^, ^102^ HBVc, P22, and CCMV VLPs have all been shown to load RNA through electrostatic interactions between the nucleic acid and the coat proteins.^25^, ^64^, ^111^, ^112^ HBVc and Qβ VLPs have also used similar principles to load DNA.^65^, ^111^, ^113^ These nucleic acid‐loaded VLPs have been developed for various uses including vaccines and vaccine adjuvants,^65^ gene delivery systems,^42^ micro RNA delivery systems,^90^, ^95^, ^102^ gene knockdown systems,^21^, ^66^ and gene replacement by delivering guide RNA for the CRISPR system.^25^ Loading and retaining nucleic acids with VLPs is easier than for small molecules because the nucleic acids are usually much larger and the capsids have evolved to load and carry similar molecules, that is, their viral genomes.

#### 5.2.3 Peptides and proteins

There are four main ways to load peptides or proteins into VLPs: (a) fusing the peptide or protein sequence to the amino acid sequence of the coat protein; (b) conjugating the peptide or protein to the genome; (c) engineering electrostatic interactions between the cargo and the coat protein; and (d) passive encapsidation. The first method loads hundreds of peptides or proteins per VLP. Both the first and second methods are facile, but have two major drawbacks. First, the peptide or protein must be amenable to genetic fusion or nucleic acid conjugation and still fold into an active form while also allowing the VLP subunits or nucleic acid to fold properly. Second, the peptide or protein must be able to exert its effect while fused to the coat proteins or conjugated to the nucleic acid. The third and fourth methods are less effective, though they allow loading of free peptides and proteins. Loading via electrostatic interactions is more effective than passive loading, but only works for peptides or proteins that have (or can be engineered to have) an affinity for the internal surface of the VLPs. HBVc VLPs have been shown to load proteins through genetic fusions either to the C‐terminus or within the protein sequence as well via passive encapsidation.^107^, ^116^ MS2 and Qβ VLPs have been used to encapsidate peptides and proteins after conjugating them to RNA containing the stem‐loop hairpin required for assembly.^21^, ^39^, ^52^, ^118^ MS2 loading has also been facilitated by electrostatic interactions between a poly‐anionic tag on the proteins and the capsid interior.^23^, ^119^ P22 has only been shown to load proteins and peptides by genetically fusing them to the scaffold protein, which in these cases is not removed from the VLP after assembly.^24^, ^25^, ^117^, ^120^, ^122^, ^123^, ^127^ CCMV loading has been accomplished using passive encapsidation, genetic fusions, and leucine zippers added to both the cargo and the coat proteins.^46^, ^62^, ^83^, ^128^ Given the difficulty in production and purification of CPMV, it is not surprising that it has not been used to load proteins yet.

#### 5.2.4 Nanoparticles

A significant body of work has studied the use of VLPs for the development of improved contrast agents. By loading the standard NP‐based contrast agents within VLPs, the new NPs gain improved relaxivities which then give higher resolution images. Additionally, if the VLPs are further modified to target specific cells, the signal‐to‐noise ratio is increased even further giving clear images of, for example, tumors. To that end, HBVc and CPMV VLPs have been loaded with iron oxide NPs through coordination to the coat proteins or through passive encapsidation.^49^, ^129^ CPMV has also been shown to load iron oxide and gadolinium NPs through coordination to the genomic RNA.^103^, ^104^ CCMV has been used to load gadolinium derivatives through electrostatic interactions with the coat proteins or “click” chemistry.^104^, ^130^ Lastly, unrelated to MRI, MS2 was loaded with quantum dot 585 for particle tracking.^21^ While not immediately therapeutically relevant, using these VLPs for diagnostics could also greatly improve patient quality‐of‐life by detecting the disease at an earlier stage and more accurately assessing therapeutic efficacy. Furthermore, iron oxide NPs have the possibility of being used for radio frequency ablation to actively destroy targeted tumor cells.^131^

### 5.3 NP uniformity and stability

Unlike the metal‐based, liposomal, and polymer‐based NPs, VLPs are highly uniform. VLPs, produced with an exact number of coat proteins and arranged in a consistent geometry, will have significantly lower lot‐to‐lot variability and identical cargo release profiles. Additionally, once inside the targeted cells, the VLPs should degrade and release all of the therapeutic cargo at once—unlike polymer NPs which slowly degrade and release the cargo over time.^75^ While slow cargo release may occasionally be beneficial, immediate release is likely to be more effective in most cases and especially for cancer treatment.

At the same time, protein‐based design means that the VLPs are not as stable as polymer NPs. Fortunately, this drawback is known and has been studied in the hopes of making better VLPs. These studies focused on HBVc, MS2, and Qβ VLPs. The HBVc VLP forms intradimer disulfide bonds that stabilize the 120 dimers, and Qβ forms disulfide bonds that link the pentameric and hexameric subunits at the 5‐ and 3‐fold axes of symmetry.^29^ A mutant MS2 VLP was also designed to form disulfide bonds within the pentamers and hexamers similar to Qβ.^29^, ^132^ Upon formation of the disulfide bond networks within these VLPs, the dissociation temperatures increased: HBVc from 72–93 to 97, MS2 from 55–70 to 73, and Qβ from 40 to 85–100°C. Furthermore, a mutant HBVc designed with an additional 240 disulfide bonds that covalently link every coat protein was engineered and shown to be stable in PBS and over multiple freeze/thaw cycles, but to disassemble in reducing conditions mimicking the cytosol.^40^ This mutant VLP shows great promise for use as a delivery vehicle.

### 5.4 Pharmacokinetics and pharmacodynamics

Although there have not been in vivo biodistribution studies for HBVc and P22 VLPs to our knowledge, in‐depth studies have been performed for MS2, Qβ, CCMV, and CPMV. We focused on studies using intravenous administration into mice or rats as model systems, which are the systems likely to be studied for initial evaluation of VLP‐based targeted therapeutics.

The distribution of MS2 VLPs, labeled internally with 64Cu or 18F, was determined in mice at 24 hr and rats at 3 hr after intravenous administration. In both cases, MS2 accumulated primarily in the liver and the spleen.^114^, ^121^ PEGylation of MS2 was also studied since PEG has been shown to act as a “stealth agent” to avoid immune clearance.^121^ PEGylated MS2 VLPs behaved similarly, except retention in the spleen was significantly reduced.^121^ This ability to avoid the immune system is extremely valuable as it will likely increase the effective dose that reaches the targeted tissue. Furthermore, work has shown that the CD47 ectodomain or the CD47 “self‐peptide,” which has been displayed on VLPs, can also be used to avoid the immune system.^106^ Qβ, labeled externally with gadolinium, was also studied in mice at 4–5 hr after intravenous administration.^97^ Qβ VLPs accumulated in the liver, but unlike MS2, accumulated at lower levels in the spleen.^97^

The biodistribution of the plant virus‐based VLPs, CCMV, and CPMV, intravenously injected in mice at various times, are mostly similar. They primarily accumulate in the liver, spleen, kidney, and GI tract.^61^, ^103^, ^133^, ^134^, ^135^ CCMV, labeled with 125I, also showed significant retention by the thyroid, probably due to the iodine.^61^ PEGylation of CPMV VLPs greatly reduced accumulation in the liver and spleen, which suggests CCMV and CPMV could also benefit from the CD47 ectodomain displayed on the surface to avoid the immune system.^106^, ^134^

Because developing VLP‐based targeted therapies for cancer is a primary application, biodistribution studies in mice possessing tumor xenografts were also conducted. MS2 or PEGylated CPMV VLPs were injected intravenously and partially accumulated in the tumors after 24 hr. This was hypothesized to be because of the EPR effect.^121^, ^135^ We suggest that the selective accumulation in these tumors could be greatly improved using cellular targeting ligands displayed on the VLPs, which was described previously, in addition to PEG or the CD47 ectodomain to avoid phagocyte engulfment.^106^

### 5.5 Specific cellular targeting and cargo delivery

**Figure 3:**
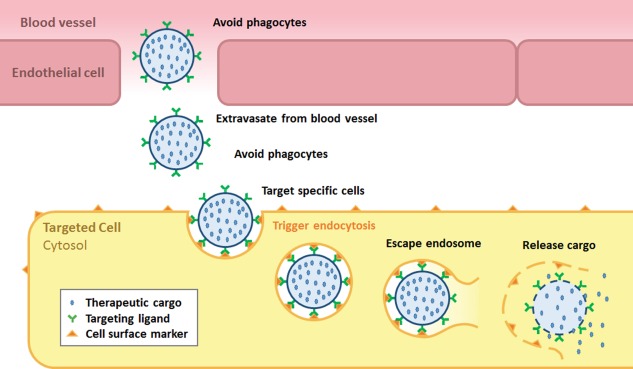
Targeted Delivery Sequence. *Stabilized VLPs* first *extravasate from the blood vessel* and then *target the specific cells* and trigger internalization while *avoiding the immune system*. Once endocytosed, the VLPs *escape the endosome* and then disassemble to *release their cargo* (italics correspond to challenges listed in Table 1)

While many different cell targeting ligands have been evaluated, ranging from glycans to specific receptor‐ligands such as folate and transferrin, the most common targeting ligand is the antibody fragment, although recently RNA and DNA aptamers have been used more frequently.^136^, ^137^, ^138^, ^139^ Most research has focused on developing ligands to target cancers, such as the anti‐HER2 antibody Trastuzumab for breast cancer and the anti‐PSMA antibodies for prostate cancer.^87^, ^88^, ^140^ However, if an effective delivery vehicle was available, it could spur research toward identifying targets on other cells, such as those involved in mitochondrial disorders and Parkinson's disease. While P22 and CCMV have not been functionalized with targeting agents, to our knowledge, the technology used to display other ligands could be easily translated for this purpose. HBVc, MS2, Qβ, and CPMV VLPs have been functionalized with antibody fragments or other targeting ligands and the targeting of most of these has been studied using cultured cells. The proposed path for these cargo delivery vehicles is outlined in Figure 3.

It has been shown that single‐domain antibody fragments could be displayed on the surface of HBVc VLPs; however, no cell targeting results have been reported.^67^ Conversely, MS2 and Qβ have been functionalized with various targeting ligands and shown to successfully target specific cells. The Matt Francis lab has functionalized MS2 VLPs with DNA aptamers with affinity for protein tyrosine kinase 7 receptors that are expressed on Jurkat leukemia T cells. They observed efficient and selective targeting of those cells by the VLPs.^84^ In addition, they modified the interior surface of MS2 with porphyrins for photodynamic therapy and demonstrated that those functionalized MS2 VLPs selectively killed Jurkat cells upon illumination.^100^ This proved that the cargo retained its functionality after delivery by the VLPs. MS2 displaying human transferrin on its surface and carrying siRNA cargo was also shown to selectively internalize into HeLa cells through receptor‐mediated endocytosis and to deliver functional siRNA.^66^ Moreover, the MS2 surface has been functionalized with a peptide (SP94) that has high affinity to human hepatocellular carcinoma cells.^21^ These SP94‐MS2 VLPs delivered their cargo, ricin toxin A‐chain, to the targeted cells and specifically killed those cells without affecting the control cells.^21^ Antibody fragments also have been displayed on the MS2 surface, although they have not been tested using cell models.^79^

Notably, the M.G. Finn group functionalized Qβ with human transferrin and observed cellular uptake and internalization of the VLPs through clathrin‐mediated endocytosis in BSC1 cells.^141^ Furthermore, they displayed glycan ligands on the Qβ surface for specific targeting of cells expressing human CD22 receptors.^85^ Those VLPs were then loaded with either green fluorescent protein or porphyrin (for photodynamic therapy) and selectively delivered to CHO cells stably expressing human CD22.^58^, ^142^ Human epidermal growth factor (EGF) as well as a fluorescent dye were displayed on the Qβ surface, and those functionalized VLPs induced autophosphorylation of the EGF receptor and apoptosis of A431 cells.^69^ In addition, as with MS2, antibody fragments have been displayed on Qβ, though no cell targeting data have been reported.^79^

Although there has not been a specific targeting study using CCMV, CCMV VLPs containing EYFP RNA were transfected into mammalian BHK cells.^143^ Those VLPs were shown to protect the RNA cargo from RNases, and EYFP expression was observed in the BHK cells.^143^ The Finn group displayed folic acid on CPMV, and showed the specific binding and endocytosis of the functionalized CPMV VLPs by KB cells expressing folic acid receptors.^86^ They also produced fluorescent dye‐labeled CPMV displaying cyclic RGD ligands to target specific integrins, and those VLPs were selectively endocytosed by several different cells overexpressing the integrins (SW480, A549, and HeLa cancer cells and HEK293 cells).^96^ Although lacking actual targeting data, the Finn group also displayed transferrin on CPMV.^144^ In addition, CPMV was functionalized with intron 8, a receptor‐binding module derived from Herstatin, to target HER2 receptors.^93^ For tumor imaging, NIR dye‐labeled CPMV VLPs were also conjugated to a bombesin analog, and their uptake by PC‐3 prostate cancer cells was observed.^58^ Tumor homing was further demonstrated using human prostate tumor xenografts on the chicken chorioallantoic membrane model.^58^ Lastly, CPMV was functionalized with a fluorescent peptide and a VEGFR‐1 specific peptide, F56, to target endothelial cells.^81^ VEGFR‐1‐targeted CPMV VLPs were shown to selectively target Ea.hy926 human endothelial cells as well as HT‐29 human colon carcinoma tumor xenografts in vivo when injected intravenously.^81^

In some cases, VLPs have been engineered to display cell‐penetrating peptides as well—either to aid in the initial cell targeting and entry or, when used in lower concentration, to aid in escaping the endosome. The use of such agents for endosomal escape may be needed to enhance the delivery of functional therapeutic cargo. After VLPs containing macromolecular cargo are endocytosed by the targeted cells, they must escape the endosome before reaching the end of the endosomal pathway: the lysosome. The lysosome will degrade the VLPs and any nucleic acid, peptide, or protein cargo they contain. Conversely, many small molecule cargoes should remain functional after VLP degradation. Previous work has displayed three different cell‐penetrating peptides on three different VLPs: MS2 has been functionalized with the HIV‐Tat peptide and a histidine‐rich H5WYG peptide, P22 has also been functionalized with the HIV‐Tat peptide, and CPMV has been functionalized with arginine‐rich R5 peptides.^21^, ^24^, ^80^, ^90^, ^95^, ^102^ One proposed mechanism of cationic cell‐penetrating peptides (HIV‐Tat and the arginine‐rich R5 peptides) is through a direct electrostatic interaction with the negatively charged phospholipids that form the endosomal membrane. This is postulated to result in membrane destabilization and endosome lysis.^31^, ^145^ Cell‐penetrating peptides containing protonatable secondary and/or tertiary amine groups (histidine‐rich H5WYG peptide) can absorb protons across the endosomal membrane, resulting in a swelling from an influx of water and/or ions and leading to rupture of the endosomal vesicle. This is known as the “proton sponge effect.”^31^ Although there are some working examples of these peptides, further research is needed.

## 6 Conclusions and perspectives

Although VLP‐based targeted drug delivery remains a nascent technology that requires further studies to prove its clinical efficacy, significant progress has been made. Many of the initial disadvantages of using VLPs have been remedied, as shown in Table 1, and the previous studies explored in this article have laid excellent groundwork for addressing the remaining challenges. Although each VLP has advantages and disadvantages relative to each other, we believe that HBVc, Qβ, and MS2 show the most promise. The advantages of these VLPs are that they:

1. can be produced using cell‐free protein synthesis^29^, ^38^
2. can load small molecules, nucleic acids, and proteins^21^, ^22^, ^52^, ^65^, ^113^, ^126^
3. can be stabilized with disulfide bonds^29^, ^40^, ^132^
4. can incorporate nonnatural amino acids for ease of surface functionalization through the “click” reaction^67^, ^79^
5. can be functionalized to display antibody fragments for specific cellular targeting^67^, ^79^
6. can be functionalized to display PEG to avoid the immune system (*not shown for HBVc VLPs*)^79^
7. will disassemble in the reducing conditions of the cytosol to release their cargo (*not shown for MS2 VLPs*)^29^, ^40^

Although it has not been experimentally proven, the disulfide bonded mutant of MS2 should behave similarly to Qβ and the disulfide bonded mutant of HBVc and disassemble in cytosolic conditions.^29^, ^40^, ^132^ Likewise, although HBVc has not been functionalized with PEG to our knowledge, the ease of nonnatural amino acid presentation and “click” conjugation will facilitate such experiments.^79^, ^101^ Additionally, all three can be functionalized with the CD47 ectodomain or the CD47 “self‐peptide” for potential avoidance of phagocytic clearance.^106^ MS2 and Qβ have also been functionalized with transferrin which may allow transcytosis across the blood‐brain barrier, allowing the VLPs to be used for neurological disorders.^66^, ^141^ While P22, CCMV, and CPMV do not currently have the same advantages as the other VLPs, we believe the same technology can be applied for them in the future.

It is also suggested that additional work focus on fully overcoming the challenges listed in Table 1. Currently, significant work has achieved loading of a variety of therapeutic cargo as well as specific cell targeting. However, efforts toward conditional VLP stabilization (including intracellular cargo release), phagocytic avoidance, and endosomal escape need to be continued. The final two relatively untouched areas where additional progress would greatly improve this technology are: (a) improving extravasation from the blood vessel to increase local concentration around the targeted cells and reduce clearance, and (b) reducing off‐target organ accumulation, mainly in the liver, kidney, and spleen. We suggest that the advances summarized here, and the suggested future directions, indicate a bright and important future for VLP‐mediated targeted drug delivery.
